# A follow-up study shows that recovered patients with re-positive PCR test in Wuhan may not be infectious

**DOI:** 10.1186/s12916-021-01954-1

**Published:** 2021-03-15

**Authors:** Xiaomin Wu, Zengmiao Wang, Zhenyu He, Yapin Li, Yating Wu, Huaiji Wang, Yonghong Liu, Fanghua Hao, Huaiyu Tian

**Affiliations:** 1 Wuhan Center for Disease Control and Prevention, Wuhan, China; 2 State Key Laboratory of Remote Sensing Science, Center for Global Change and Public Health, College of Global Change and Earth System Science, Beijing Normal University, Beijing, China; 3 Central Theater Center for Disease Control and Prevention of PLA, Beijing, China; 4 Central China Normal University, Wuhan, China

**Keywords:** COVID-19, Repeat positive, Close contact, Subsequent infection

## Abstract

**Background:**

Previous studies showed that recovered coronavirus disease 2019 (COVID-19) patients can have a subsequent positive polymerase chain reaction (PCR) test for severe acute respiratory syndrome coronavirus 2 (SARS-CoV-2) after they are discharged from the hospital. Understanding the epidemiological characteristics of recovered COVID-19 patients who have a re-positive test is vital for preventing a second wave of COVID-19.

**Methods:**

This retrospective study analyzed the epidemiological and clinical features of 20,280 COVID-19 patients from multiple centers in Wuhan who had a positive PCR test between December 31, 2019, and August 4, 2020. The RT-PCR test results for 4079 individuals who had close contact with the re-positive cases were also obtained.

**Results:**

In total, 2466 (12.16%) of the 20,280 patients had a re-positive SARS-CoV-2 PCR test after they were discharged from the hospital, and 4079 individuals had close contact with members of this patient group. All of these 4079 individuals had a negative SARS-CoV-2 PCR test.

**Conclusions:**

This retrospective study in Wuhan analyzed the basic characteristics of recovered COVID-19 patients with re-positive PCR test and found that these cases may not be infectious.

**Supplementary Information:**

The online version contains supplementary material available at 10.1186/s12916-021-01954-1.

## Term definition

Re-positive cases: The individuals who recovered from previously confirmed COVID-19 diseases and had a positive test again

Hospital stay: Time interval of a patient being hospitalized

Close contact: The individuals living in the same houses with the patients

## Background

The ongoing severe acute respiratory syndrome coronavirus 2 (SARS-CoV-2) pandemic has caused more than 108 million cases of coronavirus disease 2019 (COVID-19) illness, and over 2.4 million people globally have died from this disease as of February 2021 [1]. World Health Organization (WHO) clinical management guidelines recommend that before hospital discharge, a clinically recovered COVID-19 patient should have two sequential negative polymerase chain reaction (PCR) tests that are conducted at least 24 h apart, with testing supervised by a trained professional. However, there are reports that recovered COVID-19 patients can have a re-positive PCR test, i.e., they test positive for the virus again after two negative PCR results [2–4]. Here, we applied the definition stated in [5] where re-positive cases are those who recovered from previously confirmed COVID-19 diseases and who went on to have a subsequent positive SARS-CoV-2 PCR test. The number of patients reported to have a re-positive test is small, and the duration of follow-up has been short. Understanding the epidemiological characteristics of recovered COVID-19 patients with a re-positive PCR test is vital for preventing a second wave of COVID-19.

## Methods

### Study description

The COVID-19 outbreak was first reported in Wuhan [6] and lasted for more than 3 months; after May 18, 2020, there were no further locally acquired infections. During the outbreak in Wuhan, there were 50,340 confirmed COVID-19 cases, with 3869 deaths, and 46,471 patients were clinically cured and discharged in accordance with the WHO guidelines [7]. After being discharged from the hospital, patients in Wuhan continued to isolate in a rehabilitation center for 14 days and at home for another 14 days to prevent reinfection. These patients were regularly followed up through hospital visits. Here, the information from 118 hospitals in Wuhan on COVID-19 patients who had a positive SARS-CoV-2 PCR test between December 31, 2019, and August 4, 2020, was collected from the surveillance network of Wuhan Center for Disease Control and Prevention (Supplementary Table 1 for the names of hospitals) and performed a retrospective analysis to ascertain the epidemiological and clinical features of those with a re-positive test. Due to privacy concerns, data cannot be made openly available. For further information about the data, please contact Wuhan Center for Disease Control and Prevention.

### Data collection

COVID-19 diagnosis and illness severity was defined in accordance with the Chinese management guidelines for COVID-19 published by the National Health Commission of the People’s Republic of China. All first diagnoses of COVID-19 and all re-positive cases were confirmed by a reverse transcriptase-polymerase chain reaction (RT-PCR) test of nasopharyngeal swab or pharynx swab samples. The samples were collected and tested for SARS-CoV-2 in accordance with the WHO guidelines. The kits used are listed in Supplementary Table 2.

The discharge criteria of the recovered patients included the following: a normal body temperature for more than 3 days, obviously improved respiratory symptoms, pulmonary imaging showing obvious absorption of inflammation, and two consecutive negative SARS-CoV-2 PCR tests taken at least 24 h apart. Those who met the above criteria could be discharged. After hospital discharge, the patients continued to be in isolation for 14 days at a rehabilitation center, with health monitoring. They wore a mask and lived in a private room with good ventilation. Although the patients had contacts with the staff at the rehabilitation center, the contact was limited, and the staff wore personal protective equipment (including medical protective clothing, medical protective masks, protective goggles, medical latex gloves, shoes protectors, and work caps). The patients were followed up via regular hospital visits every 2 weeks. The patients were tested again 1 month after hospital discharge, with retests every month thereafter. Testing was organized by the local community. If the patient was positive again, he/she was transferred to the hospital or the isolation center. The patient was tested again on the 3rd–5th day after transfer and on the 11th–13th day. Individuals who had close contact with the patient were quarantined in the isolation center and were tested on the 1st–2nd day after entering quarantine and again on the 10th–14th day. Close contacts were defined as individuals living in the same houses as the patients.

### RT-PCR test

#### Nasopharyngeal swab

The sampler gently holds the person’s head with one hand, with the swab in the other, and slowly inserts the swab via the nostril so that it is deep, along the bottom of the lower nasal canal. Because the nasal canal is curved, the swab should not be forced so as to avoid traumatic bleeding. When the tip of the swab reaches the posterior wall of the nasopharyngeal cavity, it is gently rotated once (pause for a moment in case of reflex cough). It is then slowly removed, and the swab tip is dipped into a tube containing 2–3 ml of virus preservation solution (isotonic saline solution, tissue culture solution, or phosphate buffer). The swab stem is then discarded and the tube cap is tightened.

#### Pharynx swab

The sampled person first gargles with normal saline. The sampler then immerses the swabs in sterile saline (virus preservation solution is not allowed so as to avoid antibiotic allergies) and holds the person’s head up slightly. With one’s mouth wide open, making a sound “ah” to expose the lateral pharyngeal tonsils, the sampler inserts the swabs, wipes it across the tongue roots, and wipes both sides of the pharyngeal tonsils with pressure at least three times. The sampler then wipes on the upper and lower walls of the pharynxes for at least three times and dips the swab in a tube containing 2–3 ml of storage solution (isotonic saline solution, tissue culture solution, or phosphate buffer solution). The swab is then discarded the tube cap is tightened. The pharyngeal swabs can also be placed in the same tube together with the nasopharyngeal swab.

#### SARS-CoV-2 nucleic acid assay (real-time fluorescence-based RT-PCR assay)

The primers and probes used targeted the ORF1ab and N gene regions of SARS-CoV-2. Kit instructions of the manufacturers were followed for nucleic acid extraction, the real-time fluorescence-based RT-PCR reaction system, and the reaction conditions.

#### Result assessment

Negative: no Ct value or Ct value≥ 40. Positive: Ct value < 37. Gray zone: Ct value between 37 and 40; in these cases, a repeat test was recommended. If the Ct value was < 40 and the amplification curve had obvious peaks, the sample was considered to be positive; otherwise, it was considered to be negative. When a commercial kit was used, the instructions provided by the manufacturer prevailed.

### Statistical analysis

In this study, categorical variables are presented as numbers and percentages, and continuous variables are presented as the median (interquartile range, IQR). Student’s *t*-test was used to compare the continuous variables of two groups. The multivariable and univariable logistic regression model was used to determine the associated factors. All statistical analyses were performed using R version 3.4.11 (www.r-project.org). A *P* value less than 0.05 was considered to indicate statistical significance.

## Results

In total, 20,280 patients were included in the retrospective analysis. Among them, 2466 (12.16%) were re-positive for SARS-CoV-2 after they were discharged from the hospital. The demographic and epidemiological characteristics of these patients were similar to those who did not have a re-positive test (Table 1). The median hospital stay for recovered COVID-19 patients without a re-positive PCR test was 15.71 days (IQR, 9.83 to 23.21), and the median hospital stay for the original infection in the re-positive cases was 18.54 days (IQR, 11.96 to 27.04). The hospital stay for the original infection in the re-positive cases was significantly longer than that in patients without a re-positive PCR test (*P* < 0.001, Student’s *t*-test). For cases with a re-positive test, the median hospital stay of the re-positive test was 10.63 days (IQR, 7.67 to 15.63). Among the re-positive cases, the hospital stay for the re-positive test was shorter than that of the original infection (*P* < 0.001 from Student’s *t*-test). The median time from hospital discharge to the re-positive test was 11.00 days (IQR, 9.00 to 17.00). Overall, 56.12% of the re-positive cases were women. The median age of the patients was 56.00 years (IQR, 42.25 to 65.00), and more than half of the patients (50.9%, *n* = 1256) were aged between 50 and 70 years (accounting for 28.21% of the total population in Wuhan according to Wuhan Statistical Yearbook (2020) (http://tjj.wuhan.gov.cn/tjfw/tjnj/202102/t20210202_1624450.shtml)). Symptoms of the original infection for recovered COVID-19 patients with a re-positive PCR test were 0.24% asymptomatic, 49.67% mild, 35.59% moderate, 12.05% severe, and 2.40% critical. Regarding symptoms of those with a re-positive test, 2238 of 2466 re-positive cases were asymptomatic at the time of receiving a re-positive test result. Patients with asymptomatic symptoms were placed under medical observation. For those with symptoms, 193 (7.83%) patients had fever, cough, or shortness of breath. Specifically, 158 (6.41%) patients had a fever, 59 (2.39%) patients had a cough, and 18 (0.73%) patients had shortness of breath, 32 (1.30%) patients had fever and cough, 1 (0.04%) patient had cough and shortness of breath, and 9 (0.36%) patients had fever and shortness of breath. No patients had these three symptoms at the same time, and 11 of the 2466 patients passed away (not due to COVID-19).

**Table 1:** Characteristics of SARS-CoV-2 positive cases with and without a re-positive test

Characteristic	Total patients (*N* = 20,280)	Patient with re-positive test (*N* = 2466)	Patient without re-positive test (*N* = 17,814)
Hospital stay for the original infection, median (IQR)	15.96 (9.96–23.75)^5^	18.54 (11.96–27.04)^1^	15.71 (9.83–23.21)^3^
Days from discharge from hospital to re-positive test, median (IQR)	NA	11.00 (9.00–17.00)^2^	NA
Hospital stay for re-positive test, median (IQR)	NA	10.63 (7.67–15.63)^4^	NA
Sex, no. (%)			
Male	9433 (46.51%)	1082 (43.88%)	8351 (46.88%)
Female	10,847 (53.49%)	1384 (56.12%)	9463 (53.12%)
Age, no. (%)			
0~9	124 (0.61%)	6 (0.24%)	118 (0.66%)
10~19	172 (0.85%)	23 (0.93%)	149 (0.84%)
20~29	913 (4.50%)	142 (5.76%)	771 (4.33%)
30~39	2662 (13.13%)	335 (13.58%)	2327 (13.06%)
40~49	3572 (17.61%)	370 (15.00%)	3202 (17.97%)
50~59	4941 (24.36%)	588 (23.84%)	4353 (24.44%)
60~69	5319 (26.22%)	668 (27.09%)	4651 (26.11%)
70~79	1973 (9.73%)	254 (10.30%)	1719 (9.65%)
≥ 80	562 (2.77%)	80 (3.24%)	482 (2.71%)
Symptoms of first infection, no. (%)	*	*	
Asymptomatic	543 (2.68%)	6 (0.24%)	537 (3.01%)
Mild	10,304 (50.83%)	1220 (49.67%)	9084 (51.00%)
Moderate	6614 (32.63%)	874 (35.59%)	5740 (32.22%)
Severe	2365 (11.67%)	296 (12.05%)	2069 (11.61%)
Critical	379 (1.87%)	59 (2.40%)	320 (1.80%)

Cases were detected in Wuhan, China, between December 2019 and August 2020

^1^650 cases were removed owing to a lack of detailed information

^2^18 cases were removed owing to a lack of detailed information

^3^4447 cases were removed owing to a lack of detailed information

^4^1841 cases were removed owing to a lack of detailed information

^5^5097 cases were removed owing to a lack of detailed information

*10 cases were removed owing to a lack of detailed information

A logistic regression model was used to elucidate the factors related to the risk of a re-positive test. We considered three factors in the model: sex, age, and hospital stay for the original infection. Multivariable and univariable logistic regressions were performed, and the results are shown in Table 2. Both of the logistic regressions indicated that sex and hospital stay of the original infection affected the risk of a re-positive test. Men had a lower risk than women of having a re-positive test result. Recovered COVID-19 patients who had a longer hospital stay of their original infection had a higher risk of a re-positive test.

**Table 2:** Multi- and univariable regression to identify factors related to the risk of a re-positive test

Factor	Multivariable analysis		Univariable analysis	
	Coefficient	***P***-value	Coefficient	***P*** value
Age	− 0.004	*	0.002	
Gender (male)	− 0.139	**	− 0.120	**
Hospital stay for the original infection	0.023	***	0.022	***

Statistical significance: ***P < 0.001, **P < 0.01, *P < 0.05

To investigate whether re-positive cases can cause new infections in others, we investigated the PCR results of individuals who had close contact with re-positive cases. All of the close contacts were family members. Of the 2466 re-positive cases, 1201 tested positive in the rehabilitation center, and only staff wearing personal protective equipment had limited contact with them. Thus, the chance of these 1201 patients infecting others was very low. A total of 1265 patients tested positive after they returned home, and 4079 individuals had close contact with them. The PCR results were negative for all 4079 individuals. This finding indicates that these recovered COVID-19 patients with a re-positive PCR test in Wuhan may not be infectious.

## Discussion

As the COVID-19 pandemic is still going on, reports on the recovered patients with a re-positive PCR test caught public attention. Numerous studies focused on this topic [2, 4, 8–17]. Most of the literature reported the re-positive rate (from 9% (5/55) [2] to 14.5% (38/262) [4]), clinical [2, 4, 12, 14], immunological [10, 12, 14], and virological characterization of recovered patients with a re-positive PCR test [10, 12] or discussed the causes of re-positive test [11, 13]. However, the studies reporting the close contact of recovered patients with re-positive PCR test are rare. We found three studies that matched our study. The first one reported 262 COVID-19 patients and 21 close contacts from January 23 to February 25, 2020 [4]. The second one reported 119 discharged patients and 111 close contacts up till 23 April 2020 [11]. The last one reported 479 recovered COVID-19 patients and 96 close contacts from February 1 to May 5, 2020 [10]. The above reports were limited to a small group of people; the observation and follow-up times were not long enough. In our study, 20,280 COVID-19 patients and 4079 close contacts were collected from December 31, 2019, and August 4, 2020, in Wuhan. To our knowledge, this is the largest study with the longest follow-up time. Our study with these features provides more robust and accurate results. Note that our conclusion is consistent with the three studies. The current study retrospectively analyzed clinical data from a cohort of 20,280 COVID-19 patients in Wuhan. We confirmed the re-detection of SARS-CoV-2 after discharge from hospital in 12.16% of these COVID-19 patients. The time from a patient having a SARS-CoV-2 negative test to having a re-positive test ranged from 1 to 165 days, suggesting that recovered patients may still be virus carriers and require further quarantine and confirmatory negative PCR test. Note that a re-positive test could result from virus fragments coming from the original infection—the PCR test only detects fragments of the SARS-CoV-2 genome, not viable virus. A previous study of 87 re-positive cases showed that no infectious virus could be obtained by culturing, and no full-length viral genomes could be sequenced [12]. A systematic review and meta-analysis found that no studies have detected live virus beyond day 9 of illness [18, 19]. These findings may explain why there was no subsequent infection in the majority of those with a re-positive test.

There are some limitations to our study. First, we could not completely differentiate between re-positive test that were due to prolonged viral shedding of non-viable viral fragments and those that were true new infections. However, the European Centre for Disease Prevention and Control (ECDC) threat assessment brief [20] describes the full characteristics of cases of reinfection and the criteria used to identify SARS-CoV-2 reinfection. Some of these key criteria and how they relate to our findings are as follows. (1) Results from investigations of possible exposure. In our follow study, the recovered COVID-19 patients experienced two 14-days isolation periods, and the chance of viral exposure was quite low. Moreover, 84.07% (2058/2448) of the re-positive cases occurred during the 28-day isolation. (2) Time elapsed between the initial episode of infection and the suspected second episode of infection. If this duration is short, re-detection of the initial infection is a more likely than a true reinfection, because a longer time-lapse would relate to waning immunity and lower antibody levels. One study [21] showed that SARS-CoV-2 antibody levels are detectable up to 94 days after infection. In our study, the duration between hospital discharge and a re-positive test was 11.00 (IQR, 9.00 to 17.00). This indicates that the recovered COVID-19 patients may still have had antibody protection. (3) Culturing virus from multiple specimen types. If the culture is negative for viable virus, then the viral RNA detected by PCR is likely a result of non-viable viral RNA shedding rather than an ongoing infection. In this retrospective analysis, culturing results were obtained for more than 300 recovered COVID-19 patients with a re-positive PCR test and no viable virus was obtained. This finding is consistent with previous studies [12, 19]. (4) The definitions of COVID-19 reinfection, relapse, and PCR re-positivity described by Dafna Yahav et al. [22] indicate that the re-positive cases in our study were a result of PCR re-positivity rather than reinfection. Taken together, we believe that the re-positive tests resulted from viral shedding of non-viable viral fragments, and not re-infection. However, we cannot fully exclude the possibility of reinfection owing to the lack of whole-genome sequencing data and immune assessment tests even though there was a 28-day isolation period and 84.07% (2058/2448) of the re-positive cases occurred during this isolation. Nevertheless, most of these cases apparently did not cause others to become infected after discharge from the hospital.

Second, some false positives and false negatives in our study were included. The Foundation for Innovative New Diagnostics (FIND) [23] conducted independent evaluations of the 22 nucleic acid test kits for SARS-CoV-2 and reported their clinical sensitivity and clinical specificity. We used the average clinical sensitivity and clinical specificity of these 22 kits as the final sensitivity (99%) and specificity (99%). On the basis of the study by Jing Lu et al [12], the prevalence of re-positive cases in the discharged patients is 14%. Using these summarized statistics, we evaluated the effects of false positives and false negatives. The false-positive rate was 1% (false-positive rate = 1-specificity). In our study, 20,280 patients were included; therefore, the estimated number of false-positive cases is 20,280*(1–14%)*1%, which is 174.4. The false-positive cases had a small effect on the re-positivity rate. We identified 2466 re-positive cases. Of these, 1265 tested positive after returning home and having close contact with their family. The estimated number of false-positive cases in these 1265 patients is 89.5 (174.4/2466*1265). The number of true-positive cases within this 1265 patients group is 1175.5 (1265–89.5). The individuals who had close contact with these 1175.5 patients all tested negative. This indicates that no new infections were caused by the recovered COVID-19 patients with a re-positive PCR test. The false-negative rate of the RT-PCR test kits is also 1% (false-negative rate = 1- sensitivity). Thus, the number of false-negative cases would be 20,280*14%*1%, which is 28.4. This result shows that the false-negative cases had small effect on the re-positivity rate.

Third, limited patient factors were collected in this study. The results showed that sex and hospital stay of the original infection are related to having a re-positive test. Because many potential confounding factors were not analyzed, these results need to be verified using a more complete dataset.

## Conclusions

This retrospective study in Wuhan clarifies the basic characteristics of recovered COVID-19 patients with a re-positive PCR test. Furthermore, it was determined that these individuals are likely not infectious.

## Supplementary Information

**Additional file 1.**

**Additional file 2.**

## Data Availability

The data supporting the findings of this study are available within the article.

## Abbreviations

COVID-19: Coronavirus disease 2019
PCR: Polymerase chain reaction
SARS-CoV-2: Severe acute respiratory syndrome coronavirus 2
WHO: World Health Organization
RT-PCR: Reverse transcriptase-polymerase chain reaction
IQR: Interquartile range
ECDC: European Centre for Disease Prevention and Control
FIND: Foundation for Innovative New Diagnostics

## References

[1] Dong E, Du H, Gardner L (2020). An interactive web-based dashboard to track COVID-19 in real time. Lancet Infect Dis.

[2] Ye G, Pan Z, Pan Y, Deng Q, Chen L, Li J (2020). Clinical characteristics of severe acute respiratory syndrome coronavirus 2 reactivation. J Inf Secur.

[3] Lan L, Xu D, Ye G, Xia C, Wang S, Li Y (2020). Positive RT-PCR test results in patients recovered from COVID-19. JAMA.

[4] An J, Liao X, Xiao T, Qian S, Yuan J, Ye H (2020). Clinical characteristics of the recovered COVID-19 patients with re-detectable positive RNA test. Ann Transl Med.

[5] Cao S, Gan Y, Wang C, Bachmann M, Wei S, Gong J, et al. Post-lockdown SARS-CoV-2 nucleic acid screening in nearly ten million residents of Wuhan, China. Nat Commun. 2020;11. 10.1038/s41467-020-19802-w. 10.1038/s41467-020-19802-w PMC7679396 33219229

[6] Tian H, Liu Y, Li Y, Wu CH, Chen B, Kraemer MUG (2020). An investigation of transmission control measures during the first 50 days of the COVID-19 epidemic in China. Science (80- ).

[7] Organization WH (2020). Clinical management of severe acute respiratory infection (SARI) when COVID-19 disease is suspected: interim guidance.

[8] Gao Z, Xu Y, Guo Y, Xu D, Zhang L, Wang X, et al. A systematic review of re-detectable positive virus nucleic acid among COVID-19 patients in recovery phase. Infection, Genetics and Evolution. 2020;85:104494. 10.1016/j.meegid.2020.104494 PMC7403029 32763440

[9] Yuan B, Liu HQ, Yang ZR, Chen YX, Liu ZY, Zhang K (2020). Recurrence of positive SARS-CoV-2 viral RNA in recovered COVID-19 patients during medical isolation observation. Sci Rep.

[10] Yang C, Jiang M, Wang X, Tang X, Fang S, Li H (2020). Viral RNA level, serum antibody responses, and transmission risk in recovered COVID-19 patients with recurrent positive SARS-CoV-2 RNA test results: a population-based observational cohort study. Emerg Microbes Infect.

[11] Wong J, Koh WC, Momin RN, Alikhan MF, Fadillah N, Naing L (2020). Probable causes and risk factors for positive SARS-CoV-2 test in recovered patients: evidence from Brunei Darussalam. J Med Virol.

[12] Lu J, Peng J, Xiong Q, Liu Z, Lin H, Tan X (2020). Clinical, immunological and virological characterization of COVID-19 patients that test re-positive for SARS-CoV-2 by RT-PCR. EBioMedicine..

[13] Osman AA, Al Daajani MM, Alsahafi AJ (2020). Re-positive coronavirus disease 2019 PCR test: could it be a reinfection?. N Microbes N Infect.

[14] Zhu H, Fu L, Jin Y, Shao J, Zhang S, Zheng N (2020). Clinical features of COVID-19 convalescent patients with re-positive nucleic acid detection. J Clin Lab Anal.

[15] Qiao X-M, Xu X-F, Zi H, Liu G-X, Li B-H, Du X (2020). Re-positive cases of nucleic acid tests in discharged patients with COVID-19: a follow-up study. Front Med.

[16] Habibzadeh P, Sajadi MM, Emami A, Karimi MH, Yadollahie M, Kucheki M (2020). Rate of re-positive RT-PCR test among patients recovered from COVID-19. Biochem Med.

[17] Wei DH, Nian CJ, Bin PX, Ling CX, Yixian-Zhang, Fang FS (2021). Prevalence and outcomes of re-positive nucleic acid tests in discharged COVID-19 patients. Eur J Clin Microbiol Infect Dis.

[18] Cevik M, Tate M, Lloyd O, Maraolo AE, Schafers J, Ho A. SARS-CoV-2, SARS-CoV, and MERS-CoV viral load dynamics, duration of viral shedding, and infectiousness: a systematic review and meta-analysis. Lancet Microbe 2020;0. doi:10.1016/s2666-5247(20)30172-5. 10.1016/S2666-5247(20)30172-5 PMC7837230 33521734

[19] Cevik M, Bamford CGG, Ho A (2020). COVID-19 pandemic—a focused review for clinicians. Clin Microbiol Infect.

[20] Threat Assessment Brief: reinfection with SARS-CoV-2: considerations for public health response. https://www.ecdc.europa.eu/en/publications-data/threat-assessment-brief-reinfection-sars-cov-2. Accessed 6 Jan 2021.

[21] Evidence summary of the immune response following infection with SARS-CoV-2 or other human coronaviruses. 2020.

[22] Yahav D, Yelin D, Eckerle I, Eberhardt CS, Wang J, Cao B, et al. Definitions for COVID-19 reinfection, relapse and PCR re-positivity. Clin Microbiol Infect. 2020;0. 10.1016/j.cmi.2020.11.028. 10.1016/j.cmi.2020.11.028 PMC7718119 33285276

[23] FIND evaluation update: SARS-CoV-2 molecular diagnostics - FIND. https://www.finddx.org/covid-19-old/sarscov2-eval-molecular/. Accessed 15 Feb 2021.

